# DTwP-HB-Hib: antibody persistence after a primary series, immune response and safety after a booster dose in children 18–24 months old

**DOI:** 10.1186/s12887-018-1143-6

**Published:** 2018-05-28

**Authors:** Hartono Gunardi, Kusnandi Rusmil, Eddy Fadlyana, Soedjatmiko, Meita Dhamayanti, Rini Sekartini, Rodman Tarigan, Hindra Irawan Satari, Bernie Endyarni Medise, Rini Mulia Sari, Novilia Sjafri Bachtiar, Cissy B. Kartasasmita, Sri Rezeki S. Hadinegoro

**Affiliations:** 1 Department of Child Health, Faculty of Medicine, Universitas Indonesia/Dr. Cipto Mangunkusumo Hospital, Jl. Diponegoro No 71, Jakarta, 10430 Indonesia; 2 Department of Child Health, Faculty of Medicine, Padjadjaran University/Dr. Hasan Sadikin Hospital, Jl. Pasteur No 38, Bandung, 40161 Indonesia; 3 PT Bio Farma, Jl. Pasteur No 28, Bandung, Jawa Barat Indonesia

**Keywords:** Booster dose, DTwP-HB-Hib vaccine, Immunogenicity, Safety, Children

## Abstract

**Background:**

The new combination of DTwP-HB-Hib vaccines has been developed in Indonesia following World Health Organization (WHO) recommendation and integrated into national immunization program. The aims of the study were to measure 1) antibody persistence 12–18 months after a primary series, 2) immune response and safety after a booster dose of DTwP-HB-Hib.

**Methods:**

This was a multi-center, open-labeled, prospective, interventional study. Subjects who had received complete primary dose of DTwP-HB-Hib vaccine from the previous phase III trial were recruited in this trial. Subjects were given one dose of DTwP-HB-Hib (Pentabio®) booster at age 18–24 months old. Diphtheria, tetanus, pertussis, hepatitis B, *Hemophilus influenza* type B antibodies were measured before and after booster to determine antibody persistence and immune response. Vaccine adverse events were assessed immediately and monitored until 28 days after the booster recorded with parent’s diary cards.

**Results:**

There were 396 subjects who completed the study. Increased proportion of seroprotected subjects from pre-booster to post-booster were noted in all vaccine antigens: 74.5 to 99.7% for diphtheria; 100 to 100% for tetanus; 40.4 to 95.5% for pertussis; 90.2 to 99.5% for hepatitis B; and 97.7 to 100% for Hib. Common systemic adverse events (AEs) were irritability (23.7–25%) and fever (39.9–45.2%). Local AEs such as redness, swelling, and induration were significantly less common in the thigh group (7.7, 11.3, and 7.1%) than in the deltoid group (28.9, 30.7, and 25%) (*P* < 0.001). Most AEs were mild and resolved spontaneously within three-day follow-up period.

**Conclusions:**

Booster of DTwP-HB-Hib vaccine at age 18–24 months is required to achieve and maintain optimal protective antibody. The vaccine is safe and immunogenic to be used for booster vaccination.

**Trial registration:**

NCT02095314 (retrospectively registered, March 24, 2014).

## Background

Infections related with vaccine-preventable diseases including hepatitis B, diphtheria, pertussis, and *Haemophilus influenzae* type B (Hib) were accounted for high morbidity and mortality among children younger than 5 years of age in many underdeveloped countries [1–4]. In accordance with the Expanded Program on Immunization (EPI) recommendation, the Indonesian National Immunization schedule comprises primary vaccination with 3 doses of DTwP-HB-Hib at 2, 3, and 4 months, followed by a booster dose at age 18–24 months. DTwP-HB-Hib is a new vaccine produced by Bio Farma, Indonesia, combining diphtheria toxoid and tetanus toxoid, inactive pertussis bacteria, hepatitis B surface antigen, and Hib [5]. Combination vaccine reduces number of injections, number of visits to healthcare or hospital, cost, discomfort; these ultimately increase parental compliance and improve immunization coverage rates [6, 7].

In India, DTwP-HB-Hib pentavalent vaccine trial showed low reactogenicity, minimal adverse events (AEs), and high level of seroprotective rates [8, 9]. A randomized trial in Latin American children has also shown that primary and booster vaccination with a DTwP-HB-Hib combination vaccine showed good seroprotection rate and good persistence of antibodies against all vaccine antigens. The vaccine was also well-tolerated as primary and booster doses [10]. However, immunogenicity and safety of DTwP-HB-Hib combined vaccine has not been well understood in Indonesia, especially as a booster dose vaccination.

This study was a follow-up of the previous phase III study [11]. The objectives of this study were to measure antibody persistence after three primary doses at age 2,4,6 months old, to asses immune response, and to ensure safety of a booster dose of DTwP-HB-Hib vaccine.

## Methods

### Study design and population

This open-labeled, prospective, interventional and multi-center trial was conducted from March to October 2014 in Bandung (Group A) and Jakarta (Group B), Indonesia. The main criteria of subjects were children aged 18–24 months who had received hepatitis B birth dose and three primary doses of DTwP-HB-Hib vaccine from the previous Phase III trial recruited from three primary health centers in Bandung (Group A) and three primary health centers in Jakarta (Group B) [11].

Exclusion criteria in this trial were mild, moderate or severe illness, especially infectious diseases or fever (axillary temperature ≥ 37.5°C on day 0); history of allergy to any components of the vaccines; history of uncontrolled coagulopathy or blood disorders contraindicated intramuscular injection; history of acquired immunodeficiency (including HIV infection); received a treatment likely to alter immune response in the previous 4 weeks (e.g. intravenous immunoglobulin, blood-derived products or long-term corticosteroid therapy (> 2 weeks); receiving other vaccines within 1 month prior to trial enrollment; any abnormalities or chronic diseases determined by investigators that might interfere the trial objectives; and children with history of either diphtheria, tetanus, pertussis, Hib, and hepatitis B infection.

All subjects were recruited following written form of informed consent authorized by parents or legal representative after the explanation of the trial, potential risks, and his/her obligations. The study protocol had been approved by the Quality Assurance Division of Bio Farma, the Institutional Ethics Committee, and Indonesian Regulatory Authorities. This trial was conducted in accordance with ICH Good Clinical Practice guidelines and local regulatory requirement.

### Study procedure

There were two visit in the study. At the first visit, blood sample from the subjects aged 18–24 months (12–18 months after the last dose of three primary doses) were obtained for pre-booster antibody titer. Then each subject was given one dose (0.5 mL) of DTwP-HB-Hib vaccine as a booster, intra-muscularly into the middle-third anterolateral region of the thigh or the deltoid muscle with a 23G, 25 mm needle. Anterolateral thigh muscle was the preferred site but the deltoid muscle could also serve as site of injection if pediatrician considered the muscle mass was adequate.

After the booster vaccination, all parents were given a diary to record information for any local and systemic adverse events (AEs) until 28 days after the vaccination. Collection of any local or systems AEs within 3 days after immunization were conducted by a nurse or designated person by home visit or phone call.

At the second visit (28 days after the booster vaccination), blood samples were acquired from the subjects to measure post-booster antibody. Subjects’ diary were then reviewed for any notes in local and systemic AEs.

### Study vaccine

DTwP-HB-Hib vaccine (Pentabio®, batch number 5010613, with expired date: June 2015) used in this study were manufactured by Bio Farma, Bandung, Indonesia. Each 0.5 mL dose of vaccine contained ≥30 IU of purified diphtheria toxoid, ≥60 IU of purified tetanus toxoid, ≥4 IU of inactivated *Bordetella pertussis*, 10 μg hepatitis B surface antigen (HBsAg, recombinant), 10 μg Hib in the form of polyribosil-ribitol-phosphate (PRP) conjugated to tetanus toxoid, 1.5 μg aluminium phosphate, 4.5 mg sodium chloride, and 0.025 mg thimerosal. Vaccines were stored in the refrigerator at temperature + 2° to + 8°C (as standart protocol) at the clinical trial centers to assure quality.

### Blood sampling and antibody measurement

For each subject, 4 mL of blood was drawn in vacutainer tubes and then coded. After clotted at room temperature in 30 min to 2 h, each speciment was centrifuged at 3000 rpm for 15 min and the sera stored in cryotubes within 24 h after sampling. Sera of the coded samples were stored at − 20 C.

Serology antibody testing was started after the samples had been blinded. The blinding code and list were prepared by the statistician and witnessed by the investigators.

Serology assays, except for anti-HBs, were conducted in Immunology Laboratory of Product Evaluation Department of Bio Farma by technicians who were blinded to samples’ visit. Test for anti-HBs was conducted in Prodia Laboratory which had been assessed by Quality Assurance of Bio Farma and had been certificated by ISO 9001 and National Accreditation Committee. Tetanus and diphtheria antibody were measured by validated ELISA. Pertussis antibody was measured by microagglutination method. Antibody to hepatitis B surface antigen (anti-HBs) was measured by Chemiluminescent Microparticle Immunoassay (CMIA) AUSAB reagent kit by Abbott. Antibody to PRP was measured by using Improved Phipps ELISA; a competitive ELISA was used for measuring the levels of serum antibody to *Haemophilus influenzae* type B. All antibody assays were validated previously.

### Measures

This study measured antibody persistence after three doses of primary vaccinations and immune response after a single booster dose at 18–24 months of age. Antibody persistence is defined as having antibody level above the protective threshold for each given vaccination after primary doses. Immune response after booster was expressed in three parameters: (1) seroprotection (antibody level above the basic protective threshold), (2) seroconversion (conversion of seronegative to seropositive), and (3) four-times increase of antibody level [12, 13]. Primary outcome was the short-term or basic protective antibody, defined as: diphtheria antibody ≥ 0.01 IU/mL, tetanus antibody ≥ 0.01 IU/mL, pertussis antibody ≥ 1/40, hepatitis B antibody ≥10 mIU/mL, and Hib antibody titer (PRP) ≥0.15 μg/mL. Secondary outcome was long-term or full protective antibody, defined as diphtheria antibody ≥ 0.1 IU/mL, tetanus antibody ≥ 0.1 IU/mL, pertussis antibody ≥ 1/80, and Hib antibody titer (PRP) ≥1 μg/mL [14–18].

### Safety assessment

Immediate local and systemic AEs 30 min after vaccination were observed and recorded at the health centers. A digital thermometer, a plastic measuring scale, and a diary card were provided for the parents to measure and record axillary temperature and the size of redness and swelling, respectively. Appearance, duration, and intensity of any local or systemic adverse events (indicated as 1 [mild], 2 [moderate], or 3 [severe]) were recorded at home. Local reactions were defined as the presence of local pain, redness, induration, or swelling at the injection site; the systemic events were defined as the occurrence of fever (axillary temperature ≥38.0°C), irritability, or both. Local and systemic AEs were recorded in two interval of time: (1) within 30 min to 72 h and (2) 72 h – 28 days after vaccination.

Vaccine AEs were retrieved by interviewing the parents and assessing the diary card during the follow-up visit.

### Statistical analysis

The vaccine immunogenicity and safety were analyzed using intention-to-treat (ITT) and per-protocol (PP) analyses. Immunogenicity analysis was done regarding response to each vaccine antigen pre- and post-booster. Seroprotection, increase in antibody level, and seroconversion were calculated for each vaccine antigen. AEs were analyzed using Chi-square test with at least *p* value < 0.05 considered to be statistically significant.

## Results

**Fig. 1:**
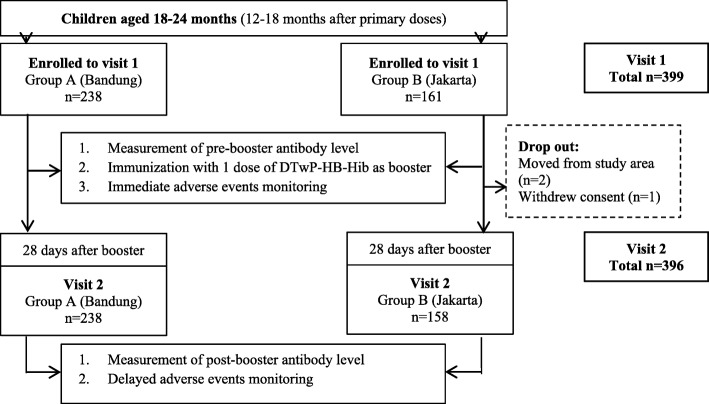
Subjects recruitment

There were 399 subjects enrolled at the first visit. These subjects were subsequently divided into two groups: of 238 children in Group A (Bandung); and 161 children in Group B (Jakarta) (Fig. 1). One subject in Group B was lost to follow-up because the child moved to another province, and there were two subjects were withdrawn from the study. After these drop-outs, the total of the subjects were 396 subjects (238 subjects in Group A and 158 subjects in Group B). All subjects were of Indonesian race, with their demographic and baseline characteristics of presented in Table 1.

**Table 1:** Demographic characteristics of subjects

Description	Group A (*N* = 238)	Group B (*N* = 161)	Total
Gender			
Male (n)	112	69	181 (45.4%)
Female (n)	126	92	218 (45.4%)
Age (months)			
Mean ± SD	19 ± 0.8	20 ± 0.9	20 ± 1.0
Min-max	18–22	19–24	18–24

### Diphtheria antibody

The protective level of diphtheria antibody was 74.5% prior the booster immunization, and 99.7% after booster. As many as 87.1% subjects had increment in their antibody titer ≥4 times, and all of seronegative subjects experinced seroconversion after the administration of booster dose. The Geometric Mean Titer (GMT) of the diphtheria antibody increased 9.4 times (0.054 to 0.508) after booster (Table 2).

**Table 2:** Summary of pre-booster and post-booster antibody level of tested antibodies^*^

Antibody	*N* = 396	Subjects (%)	95% CI	GMT(Range)
Diphtheria				
Pre-booster titer (IU/mL)				0.054 (0.047–0.061)
Anti-D ≥ 0.01^a^	295	74.5		
Anti-D ≥ 0.1^b^	81	20.5	16.8–24.7	
Post-booster titer (IU/mL)				0.508 (0.446–0.580)
Anti-D ≥ 0.01^a^	394	99.7		
Anti-D ≥ 0.1^b^	344	87.1	83.4–90.0	
Increased antibody titer^c^	345	87.1		
Seroconversion ^d^	98/98	100		
Tetanus				
Pre-booster titer (IU/mL)				0.187 (0.169–0.206)
Anti-tetanus ≥0.01 ^a^	396	100		
Anti-tetanus ≥0.1 ^b^	286	72.2	67.4–76.2	
Post-booster titer (IU/mL)				3.853 (3.531–4.205)
Anti-tetanus ≥0.01 ^a^	396	100		
Anti-tetanus ≥0.1 ^b^	394	95.5	98.2–99.9	
Increased antibody titer^c^	363	91.7		
Pertussis				
Pre-booster titer				28.086 (24.791–31.812)
≥ 1/40^a^	160	40.4	35.7–45.3	
≥ 1/80^b^	99	25.0	21.0–29.5	
Post-booster titer				639.145 (550.807–741.651)
≥ 1/40^a^	378	95.5	92.9–97.1	
≥ 1/80^b^	367	92.7	89.7–94.9	
Increased antibody titer^c^	360	90.9		
Hepatitis B				
Pre-booster titer (mIU/mL)				85.27 (74.199–98.016)
Anti-HBs ≥10	357	90.2	86.8–92.7	
Post-booster titer (mIU/mL)				5591.13 (4761.02–6564.47)
Anti-HBs ≥10	394	99.5	97.8–98.7	
Increased antibody titer^c^	385	97.2		
Seroconversion^d^	37/39	94.9		
PRP-T (Hib)				
Pre-booster titer (μg/mL)				3.399 (3.006–3.844)
Anti-Hib ≥0.15^a^	387	97.7		
Anti-Hib ≥1.0^b^	343	86.6	82.9–89.6	
Post-booster titer (μg/mL)				70.226 (61.249–80.501)
Anti-Hib ≥0.15^a^	396	100		
Anti-Hib ≥1.0^b^	394	99.5	98.2–99.9	
Increased antibody titer^c^	326	82.3		
Seroconversion^d^	9/9	100		

^*^Based on per-protocol analysis

^a^Short-term protection, ^b^Long-term protection, ^c^Increased antibody titer ≥4 times from the pre-booster level, ^d^Transition from seronegative to seropositive

### Tetanus antibody

Subjects who had the protective level of tetanus antibody were 100%, before and after the booster. However, there was an increment of GMT 20.6 times, from 0.187 to 3.853 after the booster. The proportion of subjects with 4-times increased in antibody titer was 91.7% (Table 2).

### Pertussis antibody

The subjects with pertussis micro-agglutination level ≥ 1/40 were 40.4% before booster and this number was increased to 95.5% after the booster. There were 90.9% of the subjects whose antibody titer was increased four times from the baseline, and increased of the GMT by 22.8 times, from 28.086 to 639.145, after the booster (Table 2).

### Hepatitis B antibody

Prior the booster, the proportion of subjects with protective hepatitis B antibody were 90.2, and 99.5% after booster. As many as 97.2% subjects had four times increment in their antibody titer and 94.9% subjects had seroconversion to seropositive. The GMT of hepatitis B antibody increased 65.6 times from 85.27 to 5591.13 after booster (Table 2).

### Haemophilus influenza B

The proportion of subjects with protective level of Hib antibody was 97.7% before booster and 100% after booster. As many as 82.3% subject had 4-times increment in antibody titer, and all subjects who previously were seronegative had converted to seropositive after the booster.

Overall, the GMT of Hib antibody increased 20.7 times, from 3.399 to 70.226 after booster (Table 2).

### Local adverse events 30 min after booster vaccination

Local AEs were reported in 17.3% subjects in the deltoid group and 14.9% subjects in the thigh group within 30 min after vaccination. The pain was reported in 10.8% subjects in the deltoid group and in 8.3% subjects in the thigh group. All of the symptoms were slightly less common in the thigh than in the deltoid group, but not statistically significant (*P* = 0.515).

### Systemic adverse events 30 min after booster vaccination

The most common AE was irritability, which was found in 11.7% subjects of the deltoid group and 6.5% of the thigh group.

### Local adverse events > 30 min to 72 h after booster vaccination

**Fig. 2:**
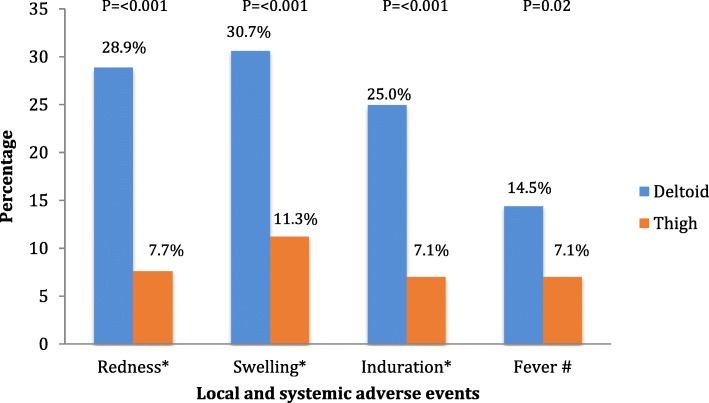
Local and systemic adverse events *local and systemic adverse events 30 minutes – 72 hours, #Systemic adverse events 72 hours – 28 days, No significant differences in AEs for pain, irritability, and others between deltoid and thigh group

Local AEs were reported in 51.3% subjects of the deltoid group and 41.7% of the thigh group within 30 min to 72 h following booster vaccination. All of the symptoms were less common in the thigh group than in the deltoid group. Pain was reported in 43.4% subjects in the deltoid group and 38.1% subjects in the thigh group. Local AEs such as redness, swelling, and induration were found significantly less common in the thigh group (7.7, 11.3, 7.1%), compared to the deltoid group (28.9, 30.7, 25%) (*P* < 0.001) (Fig. 2).

### Systemic adverse events > 30 min to 72 h after booster vaccination

There were 42.1% subjects in the deltoid group and 51.2% subjects in the thigh group who had systemic adverse events in > 30 min to 72 h after vaccination. The most common systemic AE was irritability (39.9% in deltoid group and 45.2% in thigh group), with the second most common systemic AE was fever (23.7% in deltoid group and 25% in thigh group). Both occurred slightly less common in the deltoid groups.

### Local adverse events > 72 h to 28 days after booster vaccination

No local reaction occurred within 72 h to 28 days after the booster vaccination, except 3 subjects with induration in deltoid group.

### Systemic adverse events > 72 h to 28 days after booster vaccination

There were 23.2% subjects in the deltoid group and 16.1% subjects in the thigh group who were reported to have systemic adverse events within 72 h to 28 days after vaccination. The most common symptom reported was fever (14.5% in the deltoid group vs. 7.1% in the thigh group), which statistically more significant to be found in the deltoid group (*P* = 0.02, Fig. 2). All of the other symptoms (irritability and others) were found to be slightly less common in the thigh group than in the deltoid group, but not statistically significant.

### Local and systemic reaction intensity

Most of the adverse events that were reported were mild and resolved spontaneously within the 72 h follow-up period. There was one report of acute diarrhea as a serious AE from Group B, which was classified as unrelated. The subject was recovered after several days of hospitalization. There was no other vaccine-related serious AE reported.

## Discussion

This study has demonstrated good immunogenicity and tolerability of the new combined DTwP-HB-Hib (Pentabio®) vaccine as a booster dose in children age 18–24 months old. Although the persistence of antibody following the primary doses were quite good for each vaccine antigen, there were some degrees of waning immunity during 18–24 months of age, especially diphtheria and pertussis. This justified the necessity for a booster dose in children age 18–24 months.

In a previous study, 1 month after the third dose of DTwP-HB-Hib (Pentabio®) as primary vaccination, most of the children (84–100%) had protective antibody level [11]. In this study, the antibodies prior booster vaccinations were low in subjects with protective antibody of diphtheria and pertussis (74.5 and 40.4%, respectively). After the booster, the seroprotection had increased to 99.7 and 95.5% for diphtheria and pertussis, respectively. Another DTPw-HB-Hib vaccine trial in El Salvador finds the seroconversion of *B. pertussis* after a booster dose was 94.4%. In addition, a trial in Latin America finds at least 99.1% had the seroprotective level of antibodies against diphtheria, tetanus and hepatitis B [10, 19].

Currently, there is no international standard definition determined for the seroprotection for *B. pertussis*. A study in France used the ratio 1: 80 as cut-off, but in it was stated that the cut-off might had been too high as a cut-off [20]. We used the 1/40 as cut-off, only 40.4% of our subjects had the seroprotection before the booster doses. However, this proportion had increased greatly to 95.5% after one dose of booster. The 1/40 cut-off was also used in the pertussis outbreak in a university in Japan [21]. In this study, the protective titer was found in 92.7% subjects for the long-term protection of using the 1/80 cut-off.

The persistent protective antibody after three primary doses in children aged 18–24 months was 90.2% for hepatitis B, and 97.7% for Hib. After primary immunization, anti-HBs concentrations wane quite rapidly within the first year and more slowly thereafter. Even with the waning immunity, the immune memory to hepatitis B continues to persist over a longer period. Protective antibody had risen to 99.5 and 100%; and following the booster dose, seroconversion occurred in 94.9 and 100% subjects to hepatitis B and Hib, respectively, indicating effective priming and induction of the immune memory [22].

The long-term protection of tetanus from our earlier study was 72.2% 1 month after primary DTP-HB-Hib vaccination [11], then decreased to 72.2% at 18–24 months of age. After the booster, the long-term protection had increased to 95.5% with the GMT level of 3.85 IU/mL, which will provide 3–5 years of protection [23].

DTwP-HB-Hib vaccine was found to be highly immunogenic in our booster vaccination study. One month following the booster vaccination of this vaccine, our study finds at least 95.5% of the study subjects reached protective levels of antibodies (seroprotected) against the antigens employed in the vaccine. Another report of DTwP-HB-Hib (Quinvaxem®) immunogenicity showed 99.4% seroprotection at 1-month after booster dose [24]. Other previous studies of similar pentavalent vaccine in Latin America and Costa Rica showed that it could induce both persisting immunity and boostable memory, therefore provided an efficient and reliable way of implementing this vaccine to the routine program [25, 26].

In this study, no serious adverse events were considered related to vaccine or procedure. This study has demonstrated that the occurrence of local AEs such as redness, swelling and induration within 30 min – 72 h, and fever as systemic AE in 72 h – 28 days were significantly less common in the thigh group than in the deltoid group. This findings are similar to a previous study conducted in Vaccine Safety Datalink population that included 1.4 million of children in the USA, which finds injection in thigh was associated with significantly lower risk of local reaction to DTaP vaccination among children 1–2 years of age. This finding supports the current recommendation for thigh as intramuscular site injection in this age group [27].

The three doses of primary immunization and a booster dose of DTwP-HB-Hib were all immunogenic and well-tolerated by the study subjects. DTwP-HB-Hib vaccine is a suitable for immunization program in developing countries. [9, 11, 19, 25, 26].

### Limitation of study

There were only 399 (69.4%) out of 575 subjects were recruited. A total of 396 subjects who completed the DTwP-HB-Hib primary immunization were analyzed. The reason for this limitation was due to unpredictable heavy flood in the study area, which caused so many subjects moved to other areas. Regardless the limitation, this was the first study of immunogenicity and safety of DTwP-HB-Hib booster in Indonesian children.

## Conclusions

The new combination of DTwP-HB-Hib vaccines (Pentabio) as a booster at age 18–24 months is necessary to achieve and maintain optimal protective antibody. The vaccine is safe and immunogenic to be used for booster vaccination.

## Abbreviations

DTwP: Diphtheria tetanus whole-cell pertussis
GMT: Geometric mean titer
HB: Hepatitis B
HBsAg: Hepatitis B surface antigen
Hib: *Haemophilus influenza* type b
PRP: Polyribosil-ribitol-phosphate
